# Cholesterol Screening and Statin Prescription is Low Among HIV-Infected Patients on Protease-Inhibitor Regimens in Botswana

**DOI:** 10.2174/1874613601711010045

**Published:** 2017-06-30

**Authors:** M. Mosepele, V. Letsatsi, L. Mokgatlhe, F.P. Hudson, R. Gross

**Affiliations:** 1 Faculty of Medicine, University of Botswana, Gaborone, Botswana; 2 Princess Marina Hospital, Gaborone, Botswana; 3 Department of Biostatistics, University of Botswana, Gaborone, Botswana; 4 Division of Infectious Diseases, University of North Carolina, Chapel Hill, North Carolina, USA; 5 Pearlman School of Medicine, Philadelphia, Pennsylvania, USA / Botswana-UPenn Partnership, Gaborone, Botswana

**Keywords:** Cholesterol, HIV-1 infection, Statin, Protease inhibitor, Cardiovascular risk, Sub-Saharan Africa

## Abstract

**Background:**

Little is known about the use of statin for cardiovascular disease (CVD) risk reduction among HIV-infected patients on protease inhibitors (PI`s) in sub-Saharan Africa (SSA).

**Objective:**

Cholesterol screening and statin use were retrospectively assessed among HIV-infected participants on PI`s between 2008 and 2012 at a large urban HIV clinic in Botswana.

**Methods:**

Proportion of participants screened per year was calculated and statin indication was assessed using atherosclerosis CVD (ASCVD) and Framingham risk (FRS) scores as of the year 2012 guidelines.

**Results:**

Cholesterol screening ranged between 19% and 30% per year (2008-2011) but increased to 80% after study enrollment. The rate of hypercholesterolemia (> 5.0 mmol/L) was 31% in 2012. Fewer than 1% participants were on statin therapy but 14.3% and 9.4% had statins indicated by ASCVD and FRS respectively.

**Conclusion:**

The high proportion of participants indicated for, but not prescribed statins highlights a substantial gap in the care to reduce CVD risk among these patients.

## 1 BACKGROUND

HIV-infected patients on ritonavir boosted protease inhibitor (PI) containing antiretroviral therapy (ART) are at an increased risk for elevated cholesterol. This association is the strongest among those on first generation PI`s such as ritonavir boosted lopinavir (LOP/r) [1], which is the preferred second line PI in the Botswana HIV treatment guidelines [2].

It is important to recognize and address reversible CVD risk factors such as hypercholesterolemia because HIV-infection is associated with excess risk for CVD after controlling for traditional CVD risk factors [3, 4]. Despite this established association between CVD risk and HIV-infection, adherence to CVD risk factor screening is often low among HIV providers in high resource settings [5]. Of note, hypercholesterolemia among HIV-infected patients can be effectively managed using statin therapy [6]. Additionally, statin therapy among HIV-infected patients have been associated with favourable changes in immune dysregulation such as decrease in T-cell activation and exhaustion plus lowering of biomarkers of vascular inflammation [7, 8]. However, whether cholesterol lowering and/or statin therapy is associated with decrease in CVD events among HIV-infected patients is unknown. The on-going National Institute of Health (NIH) sponsored multisite Randomized Trial to Prevent Vascular Events in HIV (REPRIEVE) will assess whether statin therapy is associated with decrease in CVD events in this population [9]. Since the results are pending from REPRIEVE, major guidelines on HIV care recommend screening for cholesterol and treating hypercholesterolemia with statins [6].

Our aim was to assess the rates of cholesterol screening plus statin use prior to study enrolment and then estimate 10 years risk of CVD among HIV-infected patients on ritonavir boosted PI’s in Botswana after enrolling patients in an observational study of cholesterol screening.

## 2 METHODS

### 2.1 Study Participants and Procedures

HIV-infected patients attending for regular care were screened for PI use at a large urban HIV clinic in Gaborone, Botswana. The study setting was one of the oldest public HIV clinics in Botswana with an estimated 8,000 adults receiving chronic HIV care, out of whom approximately 10% were on PI-containing regimens. The clinic provides HIV-relevant primary care for HIV-infected adults as recommended in the national HIV treatment guideline. The clinic offers scheduled patient visits (1-2 hourly blocks) and providers include specialist HIV nurse prescribers, medical officers and infectious diseases-trained physicians. Additional services associated with the clinic include in-house phlebotomy and a dedicated HIV pharmacy that also stocks non-HIV medicines that are routinely prescribed by the HIV clinic providers. All services are provided free of charge for all patients as part of the national HIV program.

Patients over age 21 who were on any ritonavir boosted PI containing ART were recruited by study physicians (VM, MM) during patient consultations or referred to them by other clinic providers to participate. All who agreed to participate completed written informed consent prior to participation. All participants were interviewed to ascertain risk factors for or existing cardiovascular diseases such as prior and active cigarette smoking, hypertension and diabetes mellitus and prior stroke, prior heart attack, or prior peripheral arterial disease. All interviews were conducted in a consultation room by the study physician in a language preferred by the participant (English, Setswana or a mix of both). Medical records (paper and electronic) were reviewed for any data relating to CVD risk plus HIV disease history including laboratory data and ART exposure history. All cholesterol level results between 2008 and 2012, were extracted from the medical records. Finally, all participants underwent bilateral arm blood pressure measurements and were referred for non-fasting lipid profile blood testing if they did not have a lipid profile recorded within the preceding 12 months. Participants were encouraged to complete lipid testing within 4 weeks of clinic visit, allowing the extraction of all lipid testing results to be completed within 4 weeks from the day of enrolment. Hypercholesterolemia was defined as serum cholesterol >5 mmol/L.

We used the 2013 American Heart Association/American College of Cardiologists atherosclerotic cardiovascular disease (ASCVD) risk score and Framingham Risk Score (FRS) [10, 11] to identify proportion of participants who would have been recommended statin therapy in 2012 for primary CVD risk prevention. Only active smoking status was used in the calculation of the risk scores. Because all participants were of black African origin, “African American” race was used to calculate risk scores. For the ASCVD risk score, only participants aged 40 years and older were included. Age used was the age at enrollment. The CVD risk score results are continuous measures and participants are considered to be at elevated risk for atherosclerotic cardiovascular disease if ASCVD score is equal to or greater than 7.5%, or FRS score is equal to or greater than 20%. This cut-off point thus dichotomized participants into those with elevated risk and those without, resulting in a binary variable for ASCVD. The study protocol was approved by the Ethics Board of the Botswana Ministry of Health Research & Development Committee and Committee on Human Subjects Research of the University of Pennsylvania.

### 2.2 Statistical Analysis

The proportion of participants screened for cholesterol during each year between 2008 and 2012 was calculated based on the total number of participants who were eligible for screening and were PI users during calendar year under consideration. That is, a participant was included in the denominator in the year in that he/she had PI exposure. For example, a participant who initiated PI containing ART in 2010, would only count towards the denominator during the years 2010, 2011 and 2012 but not for 2008 and 2009 (years pre-PI exposure). PI exposure was the main eligibility criterion for cholesterol screening in keeping with Botswana HIV treatment guidelines; it recommends pre-PI exposure cholesterol screening and then annual screening thereafter. All continuous data was assessed for normality and reported as means (standard deviation) if non-skewed or median (inter-quartile range) if skewed. Comparison between study groups was made using T-test for non-skewed data and Mann Whitney test for skewed continuous data. Categorical variables were compared using Chi-square tests. The kappa statistic was used to evaluate the degree of agreement between ASCVD and FRS in recommending statin therapy. Level of significance was set at p = 0.05. All the analyses were done using Statistical Package for the Social Sciences (SPSS^©^, version 22).

## 3 RESULTS

### 3.1 Baseline and Clinical Characteristics

Our clinical cohort of 375 patients was predominantly female, 239 (64%), with almost half of them, 191 (51%), with mean age of 40 or more years. Cigarette smoking was the most prevalent CVD risk factor reported among 85 (23%) participants. Other traditional CVD risk factors were less prevalent as shown in Table **1**. Among those with documented CVD related diagnosis, 31(91%), 6 (86%), 6 (67%) were prescribed medications for hypertension, diabetes mellitus and hypercholesterolemia, respectively. Only 2 (0.01%) participants reported a prior diagnosis of stroke. Females had higher cholesterol levels than men, but men reported more smoking than females.

The mean HIV disease duration for this cohort was long, 8.9 ± 2.8 years, with associated mean PI exposure duration of 4 ± 2.1 years. As shown in Table **1**, ART exposure was associated with CD4 rise and high rates of virologic suppression in excess of 90%.

### 3.2 Annual Cholesterol Screening

Fig. (**1**) shows persistently low cholesterol screening pre-study over a four year period of 2008-2011. Once enrolled in this study, the proportion of participants screened increased to 80%, in 2012, and 30% of those screened met the criteria for hypercholesterolemia.

Statin therapy was indicated for 5% more participants per the ASCVD calculator than the FRS (14% for ASCVD versus 9.4% for FRS). There was strong agreement between ASCVD and FRS in recommending statin therapy (Kappa = 0.68, 95% CI 0.54-0.82, p < 0.001).

## DISCUSSION

Our results show that provider initiated cholesterol screening among HIV-infected patients in a resource limited setting was low. When patients were enrolled in a screening program, almost 30% were diagnosed with hypercholesterolemia and 14% indicated for statin therapy according to ASCVD.

Our low screening rates were similar to what has been reported in a recent urban South African cohort. In this study by Rabkin *et al.*, only 27% of patients had been screened for elevated cholesterol [12]. With such low cholesterol screening rates, only 3% had a diagnosis of hypercholesterolemia prior to our screening intervention. However, we detected a 10 times higher prevalence (30%) of hypercholesterolemia, similar to 32% prevalence of hypercholesterolemia in focussed screening program in another urban African cohort by Julius *et al.* [13]. It is unclear why cholesterol screening rates among HIV-infected patients across clinical HIV cohorts were too low in SSA. However, we provided evidence that if recommended to be screened, patients followed using usual clinical care services. Lack of provider attention to NCD issues appears to be one of the major reasons for non-screening. Further work is required to understand patient reasons for non-screening since 20% of the study participants did not undergo screening despite it being formally recommended by study investigators. Results of such studies will provide information on the most effective strategies to increase cholesterol screening rates in this setting.

We also documented multiple reversible traditional CVD risk factors in an HIV clinical cohort. For instance, a fifth of our clinical cohort reported cigarette smoking, which is similar to the prevalence of cigarette smoking in HIV clinical cohorts in Nigeria [14], while our rates are lower than smoking rates which are in excess of 40% from North American cohorts, the higher rates in men and the difference in distribution of HIV between men and women in our setting compared to North America in part may explain this difference [15, 16]. Hypertension rates in our clinical cohort on PI`s were significantly less than 19%, 29% and 38% among other regional HIV clinical cohorts on predominantly NNRTI containing ART in South Africa and Tanzania [12, 13, 17]. Our finding of low rates of hypertension among patients on PI`s may be an effect of exclusive use of PI`s in the study group in light of a recent randomized controlled trial in which the use of lopinavir boosted ritonavir was associated with a lower risk of hypertension [18]. It is encouraging that over 90% of those with known hypertension were on treatment. However, future studies are needed to assess hypertension control in this setting.

Application of CVD risk calculators to assess statin recommendation in this clinical cohort revealed a very low use of statins among those with an indication. This finding is consonant with the lack of measurement of CVD risk factors. The higher proportion with an indication for statin therapy by ASCVD relative to FRS guidelines has also been reported among other HIV patient populations [19, 20]. Yet, there was high agreement between ASCVD and FRS in our cohort as has been reported in other small clinical HIV-cohorts [21]. Thus, the choice of tool is much less relevant than implementation of screening in general.

In summary, our study on a major urban HIV clinic in Botswana, utilizing standard clinical care procedures found that a significant proportion of patients who are recommended cholesterol screening are not being screened. However, our findings may not be generalizable to other non-urban HIV clinics across Botswana due to differences in resource availability or provider motivation. Similarly, the use of either FRS or ASCVD has not been validated in our population nor is access and understanding of use of these tools readily available across all clinics. Unfortunately, we were not able to assess factors that contributed to differences in indication for statin therapy by either risk prediction rule because of our relatively limited sample size. Furthermore, because we were not able to keep a log of all participants who were approached to participate, we were not able to accurately establish the total number of patients who were eligible for screening and also explore factors that would have resulted in those patients not getting cholesterol screening. While atazanavir is a good alternative to lopinavir to reduce the risk of hypercholesterolemia, this medication is not readily available in our setting. Thus, more research is needed to identify ways to improve cholesterol screening, and incorporate such results into a clinical risk assessment tool to identify and provide risk reduction strategies for HIV-infected who are at high risk for CVD.

## Figures and Tables

**Fig. (1):**
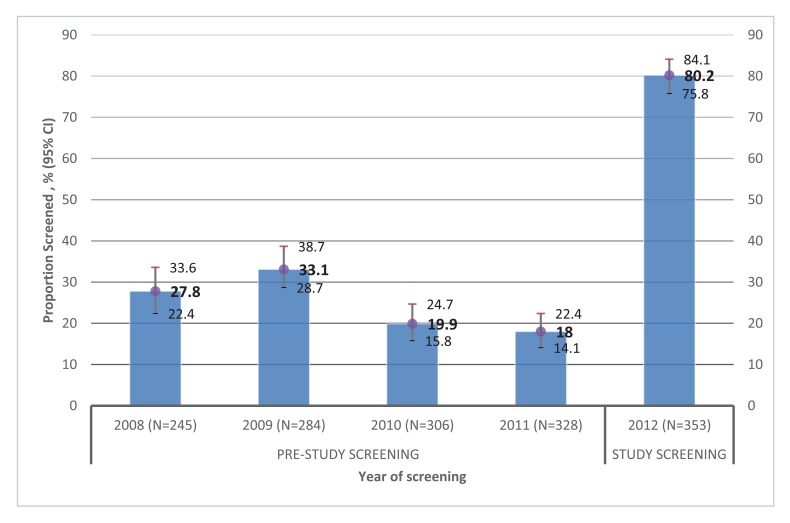
Proportion of enrollees who were screened for dyslipidemia using usual clinical practice before study enrollment (2008 to 2011) and during study enrollment (2012).

**Table 1:** Participant demographics and clinical characteristics.

	HIV-infected ART-exp*	Females	Males	^#^ *P*-value
N (%)	375	239 (63.7)	136 (26.3)	
**Demographics**				
Age Category N (%)				
21-39	167 (46.6)	131 (54.8)	36 (26.4)	< 0.01
40-49	135 (37.7)	78 (32.6)	57 (41.9)	.
50-59	41(11.5)	14 (5.9)	27 (19.9)	.
≥ 60	15 (4.2)	3 (1.3)	12 (8.8)	.
Current Cigarette Smoking, N(%)	85 (22.7)	15 (6.3)	70 (51.5)	< 0.01
Known CVD** (%)	2 (0.5)	2 (0.8)	0 (0)	> 0.9
**HIV parameters**				
Time since HIV diagnosis (years) Mean (SD)	8.9 (2.8)	8.7 (2.6)	8.9 (3.0)	0.68
^1^Cumulative ART duration (years) Mean (SD)	7.2 (2.2)	7.2 (2.2)	7.3 (2.2)	0.64
^2^Cumulative PI exposure duration (years) Mean (SD)	4.1 (2.4)	4.3 (2.3)	3.9 (2.6)	0.30
**Lopinavir N (%)**	357(95.2)	230 (96.2)	127 (93.4)	0.16
**Darunavir N (%)**	15 (4.0)	6 (2.5)	9 (6.6)	.
**Atazanavir N (%)**	2 (<0.0)	2 (0.8)	0 (0.0)	.
^3^Median Baseline CD4 count, cells/ul (IQR)	105 (126)	106 (125)	93 (130)	0.50
^4^Median CD4 nadir, cells/ul (IQR)	93 (122)	94 (122)	86 (120)	0.66
^5^Median Current CD4 count, cells/ul (IQR)	496 (389)	530 (426)	446 (321)	< 0.01
Viral load ≤ 400 copies/ml in preceding 12 months (%)	345 (92.0)	220 (92.0)	125 (92.6)	0.75
**^#^Metabolic Parameters**				
Mean Systolic pressure, mmHg (SD)	117.3 (18.8)	114.2 (17.7)	122.6 (19.6)	< 0.01
Mean Diastolic pressure, mmHg (SD)	72.9 (12.3)	72.8 (12.3)	73.1 (12.2)	0.82
Mean Total Cholesterol, mmol/L (SD)	4.6 (1.1)	4.7 (1.1)	4.5 (1.2)	0.22
Mean LDL Cholesterol, mmol/L (SD)	2.8 (0.9)	2.9 (0.9)	2.6 (0.9)	0.01
Mean HDL Cholesterol mmol/L (SD)	1.3 (0.4)	1.3 (0.4)	1.2 (0.4)	0.01
Median Triglycerides, mmol/L (IQR)	1.4 (1.1)	1.2 (0.9)	1.7 (1.6)	< 0.01
Total Cholesterol >5.0 mmol/L, N (%)	94 (31.0)	59 (24.7)	35 (25.7)	0.86
Known DM II, N (%)	7 (1.9)	2 (0.8)	5 (3.7)	0.05
Known HTN, N (%)	34 (9.1)	18 (7.5)	16 (11.8)	0.17
Known Dyslipidemia, N (%)	9 (2.4)	5 (2.1)	4 (2.9)	0.61
Statin Therapy, N (%)	6 (1.6)	2 (0.8)	4 (2.9)	0.12
HTN medications, N (%)	31 (8.3)	16 (6.7)	15 (11.0)	0.14
DM II medications, N (%)	6 (1.6)	2 (0.8)	4 (2.9)	0.12

Abbreviations: HIV, Human Immune-Deficiency Virus; ART, Anti-Retroviral Therapy; Exp, experienced; CVD, cardiovascular disease; PI, Protease Inhibitor; CD4, cluster of differentiation 4; LDL, low Density Lipoprotein; HDL, High Density Lipoprotein; DM II, Diabetes Mellitus II; HTN, Hypertension

*Means (standard deviation) for normally distributed continuous measures, medians (IQR) for non-normally distributed continuous measures, counts (percentages) for categorical variables

**Stroke, Heart attack & Peripheral arterial diseases

^1^Cumulative ART duration; ART prescription since initial date of enrollment

^2^Cumulative PI exposure duration; PI prescription since initial script until enrollment

^3^Baseline CD4 count; last CD4 count recorded prior to ART initiation

^4^CD4 nadir; lowest recorded CD4 count prior to any ART exposure

^5^Current CD4 count; most recent CD4 available in the medical records

^#^Measurement at time of enrollment in 2012
